# Source-directed antimicrobials: a shot in the dark?

**DOI:** 10.1186/cc10663

**Published:** 2012-03-20

**Authors:** L Richardson, GB McNeill, S Gupta

**Affiliations:** 1 University Hospital Southampton NHS Foundation Trust, Southampton, UK; 2 Sheffield Teaching Hospitals NHS Foundation Trust, Sheffield, UK

## Introduction

The Surviving Sepsis Campaign advocates giving early empirical antibiotics directed against all likely pathogens [1]. The failure to instigate antimicrobials against a later confirmed pathogen impacts negatively on mortality [2]. Many hospitals advise source-directed therapy from the beginning. Our project aims to elicit the proportion where the source of sepsis is initially predicted incorrectly thereby putting patients at risk.

## Methods

A prospective cohort study was performed in two UK teaching hospitals of patients presenting with sepsis to critical care between May 2010 and March 2011. Hospital computer systems and patient notes were used to extract the initial suspected source of sepsis, and later verified with true microbiology data. Overall mortality was measured and compared between correctly and incorrectly suspected source of sepsis patients.

## Results

**Figure 1:**
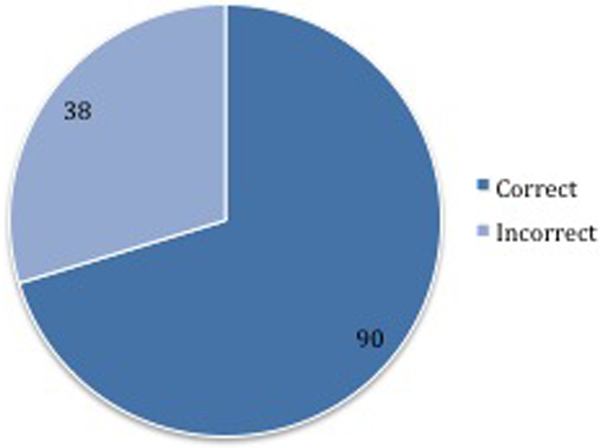
**Proportion of correctly and incorrectly identified sources**.

Of the 128 patients, the source of sepsis was wrongly identified in 30% (38/128) (Southampton 28% 15/53, Sheffield 31% 23/75 respectively) (Figure 1). The most common source was the bowel, which was initially suspected as a respiratory source in most cases. Interestingly, the mortality was higher in the correctly identified group (13%, 16/128 vs. 5%, 7/128). This probably reflects the severity of illness where the diagnosis is sometimes more obvious.

## Conclusion

Good antimicrobial governance requires early administration of narrow-spectrum antibiotics as best guess source-directed therapy from the outset, because de-escalation is often not practical. Our data reveal that in 30% of cases we incorrectly guess the source.

We advise that in patients with severe sepsis or septic shock first-line antibiotics should remain broad spectrum with rigorous follow up to de-escalate as early as possible.
